# Female Doctors in Philadelphia

**Published:** 1882-06

**Authors:** 


### FEMALE DOCTORS IN PHILADELPHIA.

The action of the Philadelphia County Medical Society in refusing recently to receive into membership five ladies who applied for admission, has been the subject of considerable comment on the part of the medical press of the country. These women were regular and full-fledged graduates, it seems, of a medical college in good standing, and were actively engaged in the practice of medicine in Philadelphia. Their applications for membership were indorsed and recommended by the board of censors of the society. But for all this they were enthusiastically and no doubt very properly rejected. We are not fully informed as to the motives which induced these gentlemen to avail themselves so promptly of their undoubted prerogative of selecting their own associates. It was their own affair and they undoubtedly had a perfect right to determine the question as they saw fit, without vouchsafing any explanation whatever, and we only refer to the matter at all to say that if this decision of the Philadelphia County Medical Society shall be accepted by women as a discouragement from entering the ranks of the medical profession it will have done them a real service, and the act will be confirmed as not only wise but patriotic.

There are better and more appropriate fields of labor, and usefulness for women than can be found in any of the so called learned professions, and the sooner they come to a full recognition of this fact the fewer disappointments will they experience, and fewer will be the instances of misguided ambition annually recorded.

